# ROS-Responsive miR-150-5p Downregulation Contributes to Cigarette Smoke-Induced COPD via Targeting IRE1*α*

**DOI:** 10.1155/2022/5695005

**Published:** 2022-05-05

**Authors:** Mengchan Zhu, Ling Ye, Guiping Zhu, Yingying Zeng, Chengyu Yang, Hui Cai, Yuqing Mo, Xixi Song, Xin Gao, Wenjun Peng, Jian Wang, Meiling Jin

**Affiliations:** Department of Pulmonary and Critical Care Medicine, Zhongshan Hospital, Fudan University, Shanghai, China

## Abstract

MicroRNAs (miRNAs) have been reported in human diseases, in which chronic obstructive pulmonary disease (COPD) is included. Herein, we assessed the role along with the possible mechanisms of miR-150-5p in cigarette smoke- (CS-) induced COPD. The plasma miR-150-5p expression was lower in patients with COPD and acute exacerbation of COPD (AECOPD) and was related to disease diagnosis, disease severity, and lung function. Consistently, exposure to CS for 3 months or 3 days reduced miR-150-5p in the plasma and lung tissues of mice, and CS extract (CSE) inhibited miR-150-5p in human bronchial epithelial cells (HBECs) in a concentration along with time-dependent approach. In vitro, miR-150-5p overexpression decreased the contents of inflammatory factors interleukin- (IL-) 6, IL-8 along with cyclooxygenase-2 (COX-2), and endoplasmic reticulum (ER) stress markers glucose-regulated protein (GRP) 78 and C/-EBP homologous protein (CHOP) and promoted cell migrate. Mechanistically, miR-150-5p could bind with the 3′-untranslated region (UTR) of inositol requiring enzyme 1*α* (IRE1*α*), while IRE1*α* overexpression obliterated the impacts of miR-150-5p. Besides, N-acetyl-cysteine (NAC) reversed CSE-induced miR-150-5p downregulation and its downstream effects. In vivo, miR-150-5p overexpression counteracted CS-triggered IRE1*α* upregulation, inflammation, and ER stress in the lung tissues of mice. In conclusion, our findings illustrated that ROS-mediated downregulation of miR-150-5p led to CS-induced COPD by inhibiting IRE1*α* expression, suggesting to serve as a useful biomarker for diagnosing and treating COPD.

## 1. Introduction

Chronic obstructive pulmonary disease (COPD), which is characterized by respiratory manifestations and progressive airflow obstruction, contributes significantly to global morbidity and mortality [1]. Nearly three million people die of this disease each year, accounting for 6% of all deaths worldwide [2]. According to the latest China Pulmonary Health study, COPD prevalence has risen to 13.7% among people aged 40 years and above [3]. Despite the fact that plenty of factors contribute to increasing the risk of developing COPD, cigarette smoke (CS) exposure remains the primary cause [4, 5]. However, the mechanisms underlying CS-induced COPD pathogenesis are still unclear, which has limited the development of therapies for these patients.

MicroRNAs (miRNAs) are noncoding RNA molecules of ~22 nucleotides and control gene expression negatively by either degrading target messenger RNA (mRNA) or inhibiting protein translation [6, 7]. As reported, miRNAs are linked with the onset and progress of numerous diseases, such as COPD. More importantly, miRNAs can be stably present in various body fluids, emerging as promising biomarkers for disease diagnosis and prognosis evaluation [8]. We have previously utilized the GEO database to construct a blood miRNA-mRNA regulatory network in COPD patients [9]. Recently, circulating miR-150-5p in many cancers has been extensively studied [10–13], but not limited. For example, reduced serum miR-150-5p levels were related to unfavorable outcomes in critical illness patients [14]. In COPD, low blood miR-150-5p levels were associated with poor survival after disease diagnosis [15]. Besides, miR-150-5p has also been found to protect against acute CS or CS extract- (CSE-) induced lung inflammation and cell apoptosis [16]. To date, there have been no further research reports evaluating the role along with potential mechanisms of miR-150-5p in COPD pathogenesis.

Inositol requiring enzyme 1*α* (IRE1*α*) constitutes the most evolutionarily conserved sensor protein during endoplasmic reticulum (ER) stress, possessing both kinase and RNase activities [17, 18]. Under ER stress, IRE1*α* autophosphorylates, as well as activates its RNase domain to catalyze the splicing of X-box binding protein 1 (XBP1) mRNA to trigger adaptive signal transduction, termed unfolded protein response (UPR). In addition, IRE1*α* can also degrade many ER-localized mRNA and precursors of apoptosis-inhibitory miRNAs by a regulated IRE1*α*-dependent decay (RIDD) process [19]. Selective activation or inhibition of IRE1*α* has proven promising for disease treatment [20]. A previous study reported that IRE1*α* was upregulated in sarcopenic COPD patients [21]. However, it remains to be determined about the role of IRE1*α* in COPD pathogenesis.

Herein, we demonstrated the downregulation of miR-150-5p in COPD patients, CS-exposed mice, and CSE-stimulated human bronchial epithelial cells (HBECs). miR-150-5p overexpression reduced CSE or CS-induced inflammation and ER stress and promoted cell migration by directly targeting IRE1*α* in vitro and in vivo. Besides, reactive oxidative species (ROS) modulated CSE-induced miR-150-5p downregulation and its downstream effects. These findings may be useful in understanding the pathogenesis of COPD and developing precision medicine for its treatment.

## 2. Materials and Methods

### 2.1. Study Subjects

The inclusion and exclusion criteria were previously reported [22]. Briefly, for COPD patients, these are as follows: (1) age ≥ 40 years, (2) forced expiratory volume in 1st second to forced vital capacity (FEV_1_/FVC) is less than 70% after bronchodilator treatment, and (3) stable disease upon enrollment. The term “acute exacerbation of COPD” (AECOPD) referred to a sudden worsening of respiratory symptoms in COPD patients that needed further treatment. As the control subjects, age- and sex-matched nonsmokers and smokers without COPD were included. COPD patients, AECOPD patients, and normal controls were excluded if they had (1) bronchiectasis; (2) tuberculosis; (3) hospitalization for a cardiac condition or thoracic and abdominal surgery within the past month; (4) mental illness, dementia, or impaired comprehension; (5) stroke and high paraplegia; (6) tumor; and (7) pregnancy or breastfeeding. This research work was approved by the Ethics Committees of Zhongshan Hospital, Fudan University (Approval ID: B2017-022R). Written informed consent was signed by each subject before participation in the study.

### 2.2. Mice and CS Exposure

Male C57BL/6 mice (6-8 weeks) were commercially acquired from JSJ laboratories (Shanghai, China) and kept at the animal facilities of Zhongshan Hospital. For the 3-month CS-exposed mouse model, exposure of the mice to CS was performed in a stainless-steel chamber using a whole-body smoke exposure system with 20 cigarettes, two times per day, six days per week. For the 3-day CS-exposed mouse [16, 23], we exposed the mice to CS for 50 cigarettes, five times per day. The effects of miR-150-5p on lung injury induced by CS were evaluated with administering miR-150-5p agomiR (agomiR-150-5p) and its negative control (agomiR-NC; 10 nM per mouse; GenePharma, Shanghai, China) nasally to mice, followed by 3-day CS exposure. We exposed the mice of the control group to room air. Posttransfection, the last CS exposure 24 h, all mice were sacrificed. The Animal Care and Use Committee of Zhongshan Hospital ethically examined and approved all animal procedures.

### 2.3. CSE Preparation

As previously described [24, 25], the CSE was prepared as follows: five cigarettes (Daqianmen, Shanghai, China) were burned consecutively and bubbled through an experimental apparatus containing 10 mL RPMI-1640 medium (Gibco, Waltham, MA, USA). The CSE obtained was sterile filtered and served as 100% CSE when used.

### 2.4. Cell Culture and Treatment

HBECs were acquired from Chinese Academy of Sciences and grown with RPMI-1640 medium that contains 10% FBS (Gibco) at 37°C, in a 5% CO2 incubator. 4-Phenyl butyric acid (4-PBA; 5 *μ*M or 10 *μ*M; MCE, Monmouth Junction, NJ, USA) or N-acetyl-cysteine (NAC; 5 mM; Beyotime, Shanghai, China) was used to treat HBECs 1 h prior to 5% CSE stimulation.

### 2.5. Cell Transfection

The miR-150-5p mimic, IRE1*α* small interfering RNA (siRNA), IRE1*α* overexpression vector, and their controls were purchased from GenePharma and transfected into HBECs using Lipo8000 reagent (Beyotime). 24 h posttransfection, we stimulated the cells with 5% CSE.

### 2.6. Wound Healing Assay

HBECs were seeded into 12-well plates and incubated to reach confluence. Draw a straight line through the cells along a ruler, and the plates were washed with PBS three times for removing debris. To minimize effects of cell proliferation [26, 27], the cells were then cultured in RPMI-1640 medium enriched with 2% FBS and were photographed with a light microscope (Olympus, Tokyo, Japan).

### 2.7. Real-Time Quantitative PCR (RT-qPCR)

RNA isolation from plasma was done with TRIzol-LS reagent (Invitrogen), but from HBECs along with lung tissues using TRIzol reagent (Invitrogen). Generation of cDNA from mRNA along with miRNA was done with the PrimeScript RT Master Mix (TaKaRa Bio, Shiga, Japan) and miRNA First Strand cDNA Synthesis Kit (Sangon Biotech, Shanghai, China), respectively. RT-qPCR was run with TB Green Premix Ex Taq (TaKaRa Bio) on a Bio-Rad system (Hercules, CA, USA). mRNA along with miRNA was quantified via the established 2^-△△Ct^ approach with GAPDH and U6 as endogenous controls, respectively. Primer sequences are given in Supplementary Table 3.

### 2.8. Western Blot

Protein was prepared with radioimmunoprecipitation assay (RIPA; Beyotime) buffer. 10% SDS-PAGE (Epizyme, Shanghai, China) was used to separate equal amounts of protein. Transferred membranes were blocked and incubated at 4°C overnight with primary antibodies against COX-2 (#12282; CST, Danvers, MA, USA), GRP78 (#3177; CST), CHOP (#2895; CST), IRE1*α* (#3294; CST), GAPDH (AF1186; Beyotime) and *β*-actin (AF0003; Beyotime). Following three washes with TBST, incubate secondary antibodies (Beyotime) on the membranes for 1 h at room temperature. With enhanced chemiluminescence (ECL) reagents (Beyotime), the antibody-bound bands were visualized on a Tanon 5200 Multi (Tanon, Shanghai, China) and quantified using the ImageJ software (version 1.46r).

### 2.9. Luciferase Reporter Assay

Based on the RNA22 database [28], miR-150-5p bound to IRE1*α* in the 3′-untranslated region (UTR). The dual-luciferase reporter vector containing wild type (WT) or mutant type (MUT) IRE1*α* 3′-UTR was constructed by Obio Technology Corp., Ltd. (Shanghai, China) and cotransfected with miR-150-5p mimic or NC mimic into HBECs. Luciferase activity was measured using a dual-luciferase reporter assay system (Beyotime) 48 h after transfection.

### 2.10. ROS Detection

The intracellular ROS level was measured with a dichlorodihydro-fluorescein diacetate (DCFH-DA) fluorescence kit (Beyotime). HBECs were treated with NAC prior to 5% CSE stimulation and then loaded with DCFH-DA probes (10 *μ*M) diluted in RPMI-1640 medium. Following incubation at 37°C for 20 mins, three washes with RPMI-1640 medium were performed. The generation of ROS was observed under a fluorescence microscope (Olympus) and quantified with the ImageJ software (version 1.46r).

### 2.11. Hematoxylin and Eosin (H&E) Staining

The left upper lungs were fixed (in 4% PFA) and then paraffin-embedded, followed with sectioning for H&E staining, as described by the manufacturer.

### 2.12. Fluorescence In Situ Hybridization

After deparaffinization, the lung tissue sections were blocked with prehybridization buffer at 37°C for 1 h and then incubated with miR-150-5p probe synthesized by Servicebio (Wuhan, China) in hybridization buffer at 4°C overnight. Following three washes with PBS, the nuclei were stained for 5 mins with DAPI. Capturing of the fluorescent images was done with a fluorescence microscope (Olympus).

### 2.13. Immunohistochemistry

Sections of lung tissue were deparaffinized and blocked and then incubated with anti-IRE1*α* antibody (1 : 100 dilution), followed by PBS wash, secondary antibody incubation, and DAB staining. Immunohistochemical images were captured under a light microscope (Olympus).

### 2.14. Statistical Analysis

Data are given as mean ± SEM and analyzed in the GraphPad Prism 7. Comparison of two groups was done utilizing *t*-test, whereas comparison of three or more groups was achieved by using ANOVA. Pearson's correlation analysis was applied for analyzing the association between plasma miR-150-5p expression and lung function indices FEV_1_, FEV_1_% predicted (FEV_1_%pred), and FEV_1_/FVC and lung miR-150-5p expression. A *P* < 0.05 was considered significant.

## 3. Results

### 3.1. miR-150-5p Is Downregulated in COPD Patients and CS-Exposed Mice

**Figure 1:**
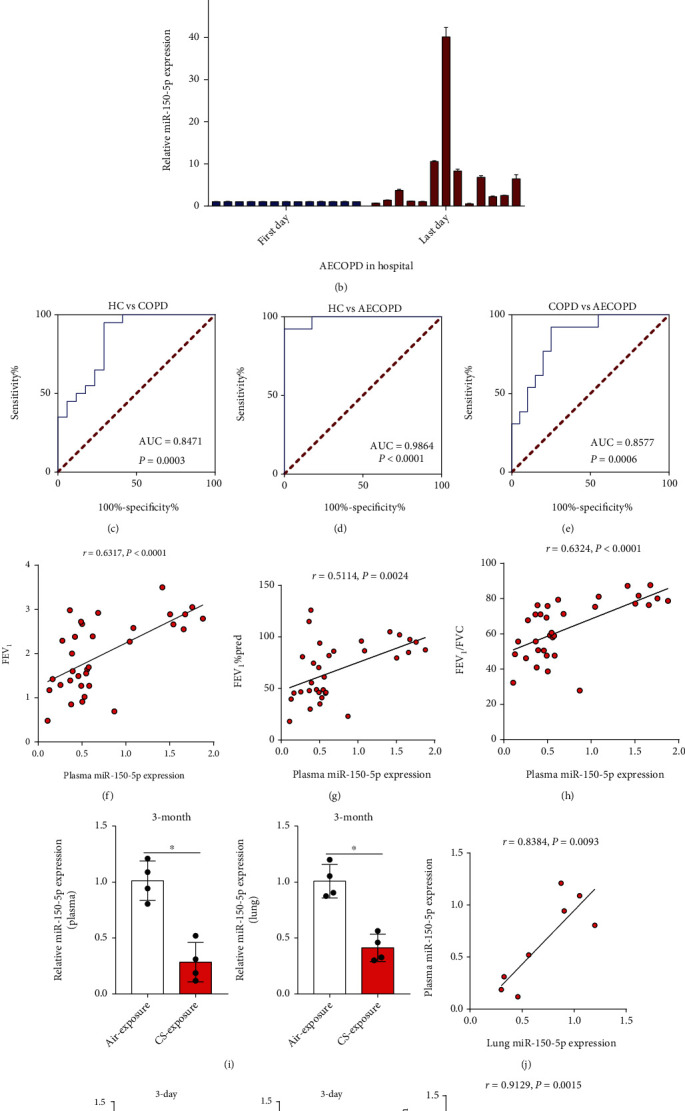
miR-150-5p is downregulated in COPD patients and CS-exposed mice. (a) RT-qPCR assessment of miR-150-5p expression in the plasma of nonsmokers (*n* = 7), smokers (*n* = 10), COPD patients (*n* = 20), and AECOPD patients (*n* = 13). (b) RT-qPCR assessment of miR-150-5p expression in the plasma of AECOPD patients on the first day and last day of hospitalization. ROC curves for plasma miR-150-5p to distinguish between (c) normal controls and COPD patients, (d) normal controls and AECOPD patients, and (e) COPD and AECOPD patients. Correlation assessment of plasma miR-150-5p expression and (f) FEV_1_, (g) FEV_1_%pred, and (h) FEV_1_/FVC. (i) RT-qPCR assessment of miR-150-5p expression in the plasma and lung tissues of 3-month CS-exposed mice. (j) Association between miR-150-5p expression in plasma and lung tissues in 3-month CS-exposed mice. (k) RT-qPCR assessment of miR-150-5p expression in the plasma and lung tissues of 3-day CS-exposed mice. (l) Association between miR-150-5p expression in plasma and lung tissues in 3-day CS-exposed mice. ^∗^*P* < 0.05.

Stable expression of miRNAs in body fluids is essential for their use as diagnostic biomarkers. First, we searched for miR-150-5p expression in different samples through the YM500v2 database [29] and found that it was highly abundant in the blood (Figure S1(a)). In the GSE31568 dataset, blood miR-150-5p was downregulated in COPD patients (*n* = 70) than normal controls (*n* = 24; Figure S1(b)). After that, we validated miR-150-5p expression in plasma from seven nonsmokers, ten smokers, 20 COPD patients, and 13 AECOPD patients, and subjects' characteristics are shown in Supplementary Table 1. In comparison to nonsmokers and smokers, plasma miR-150-5p was reduced in COPD patients and further reduced in AECOPD patients (Figure 1(a)). In addition, we analyzed whether plasma miR-150-5p expression in the above 13 AECOPD patients was changed during hospitalization, and detailed treatments of these patients are shown in Supplementary Table 2. For these AECOPD patients, plasma miR-150-5p expression on the last day of hospitalization was higher than that on the first day of hospitalization, suggesting that miR-150-5p in plasma might be linked with disease severity (Figure 1(b)). ROC curve analysis exhibited that miR-150-5p in plasma had diagnostic values for differentiating COPD patients (AUC value = 0.8471, *P* = .0003; Figure 1(c)) and AECOPD patients (AUC value = 0.9864, *P* < .0001; Figure 1(d)) from normal controls and AECOPD patients from COPD patients (AUC value = 0.8577, *P* = .0006; Figure 1(e)). Pearson's correlation assessment demonstrated that miR-150-5p in plasma was positively correlated with lung function indices FEV_1_ (*r* = 0.6317, *P* < .0001; Figure 1(f)), FEV_1_%pred (*r* = 0.5114, *P* = .0024; Figure 1(g)), and FEV_1_/FVC (*r* = 0.6324, *P* < .0001; Figure 1(h)).

Moreover, we validated levels of miR-150-5p in two CS-exposed mouse models: a 3-month CS-exposed mouse model (chronic exposure) and a 3-day CS-exposed mouse model (acute exposure). As illustrated in Figures 1(i) and 1(k), both plasma and lung tissues showed reduced expression of miR-150-5p in the two different models. Importantly, there was a remarkable positive relationship of plasma miR-150-5p downregulation with lung miR-150-5p downregulation in the 3-month CS-exposed mice (*r* = 0.8384, *P* = .0093; Figure 1(j)), as well as in the 3-day CS-exposed mice (*r* = 0.9129, *P* = .0015; Figure 1(l)). The above results illustrated that miR-150-5p may be a meaningful indicator for COPD.

### 3.2. CSE Downregulates miR-150-5p Expression, Induces Inflammation and ER Stress, and Reduces Cell Migration in HBECs

**Figure 2:**
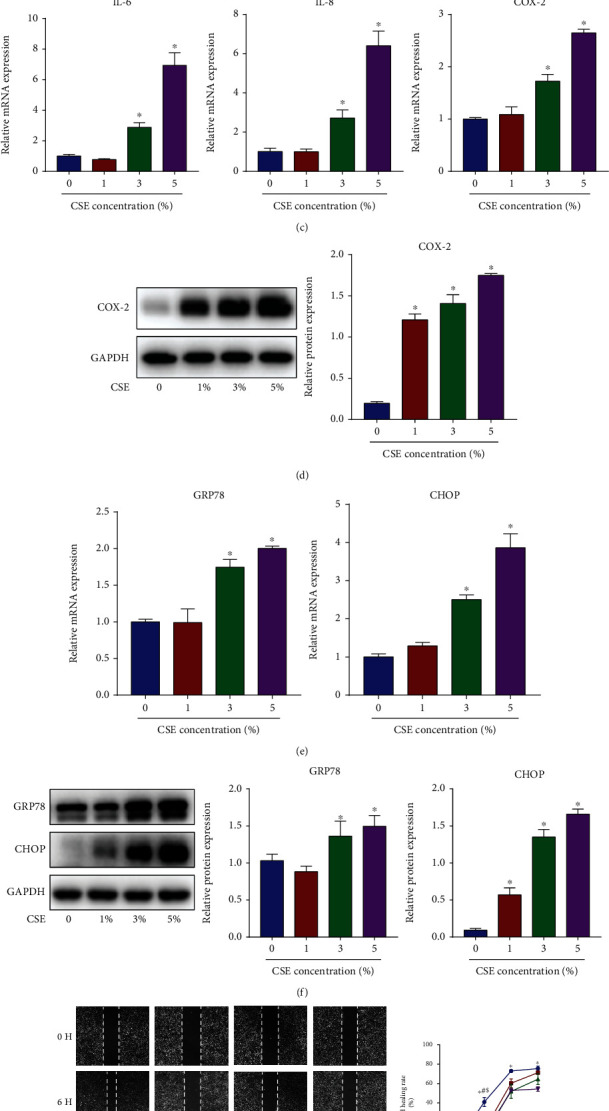
CSE downregulates miR-150-5p expression, induces inflammation and ER stress, and reduces cell migration in HBECs. RT-qPCR assessment of miR-150-5p expression in HBECs exposed to (a) diverse levels of CSE (1, 3, and 5%) for 24 h or to (b) 5% CSE for different times (6, 12, and 24 h). HBECs were exposed to with diverse levels of CSE (1, 3, and 5%) for 24 h. (c) RT-qPCR assessment of IL-6 and IL-8 along with COX-2 mRNA contents in HBECs. (d) Western blot images and quantification of COX-2 protein contents in HBECs. (e) RT-qPCR assessment of GRP78 and CHOP mRNA contents in HBECs. (f) Western blot images and quantification of GRP78 and CHOP protein contents in HBECs. (g) Image analysis of cell migration in HBECs exposed to diverse levels of CSE (1, 3, and 5%) for different times (6, 12, and 24 h). ∗, #, and $ indicate *P* < 0.05; *n* = 3.

Airway epithelial injury is the pathological basis for the development of COPD, and thus, HBECs were cultured and stimulated with CSE as an in vitro model. In HBECs, CSE decreased miR-150-5p expression concentration-dependently (Figure 2(a)) and time-dependently (Figure 2(b)). Also, CSE elevated the contents of inflammatory factors IL-6, IL-8 along with COX-2 (Figures 2(c) and 2(d)), and ER stress markers GRP78 and CHOP (Figures 2(e) and 2(f)) and suppressed cell migration in a concentration and time-dependent approach (Figure 2(g)). These results suggested that CSE could reduce miR-150-5p expression and induce airway epithelial injury by promoting inflammation and ER stress and inhibiting cell migration.

### 3.3. miR-150-5p Inhibits Inflammation and ER Stress and Promotes Cell Migration in CSE-Stimulated HBECs

**Figure 3:**
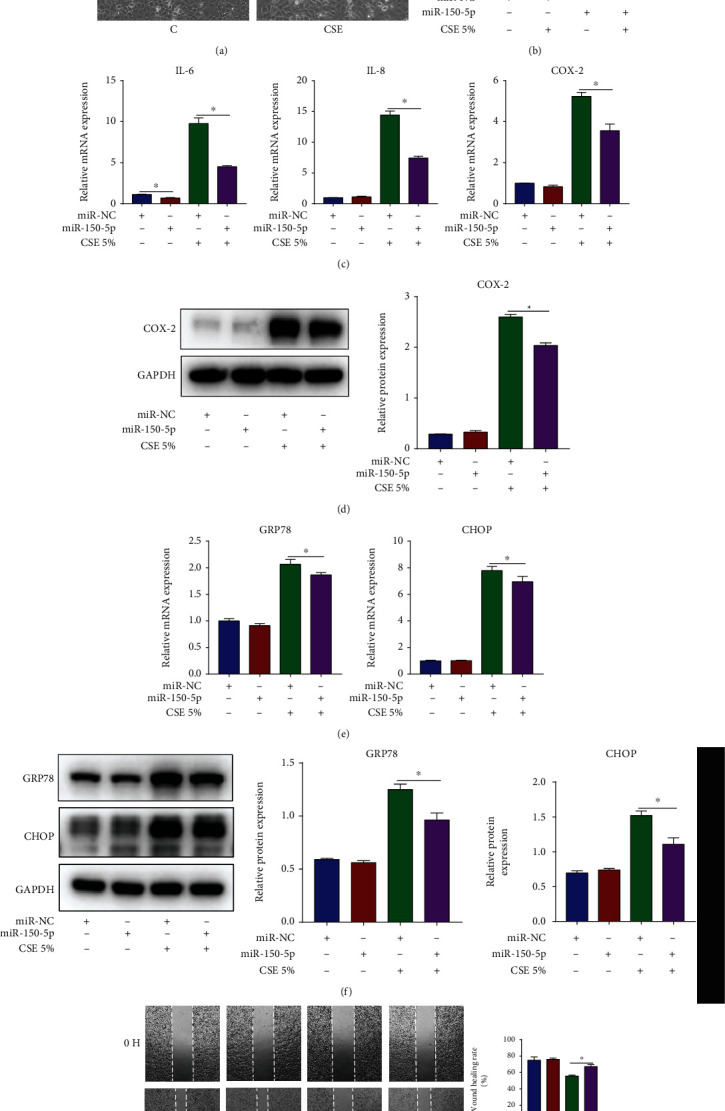
miR-150-5p ameliorates inflammation and ER stress and promotes cell migration in CSE-stimulated HBECs. HBECs were transfected with miR-150-5p mimic or NC mimic and then exposed to 5% CSE for 24 h. (a) Cell morphology. (b) RT-qPCR assessment of miR-150-5p expression. (c) RT-qPCR assessment of IL-6 and IL-8 along with COX-2 mRNA contents in HBECs. (d) Western blot images and quantification of COX-2 protein contents in HBECs. (e) RT-qPCR assessment of GRP78 and CHOP mRNA contents in HBECs. (f) Western blot images and quantification of GRP78 and CHOP protein contents in HBECs. (g) Image analysis of cell migration. ^∗^*P* < 0.05; *n* = 3.

To demonstrate miR-150-5p's effects on CSE-induced airway epithelial injury, miR-150-5p mimic was utilized to upregulate miR-150-5p in HBECs before 5% CSE stimulation. Cell morphology and miR-150-5p expression after transfection are shown in Figures 3(a) and 3(b), respectively. In CSE-stimulated HBECs, miR-150-5p mimic decreased the contents of IL-6, IL-8 along with COX-2 (Figures 3(c) and 3(d)), and GRP78 and CHOP (Figures 3(e) and 3(f)) and promoted cell migration (Figure 3(g)). The above results illustrated that miR-150-5p was a protective miRNA in CSE-induced airway epithelial injury.

### 3.4. ER Stress Regulates Inflammation and Cell Migration in CSE-Stimulated HBECs

**Figure 4:**
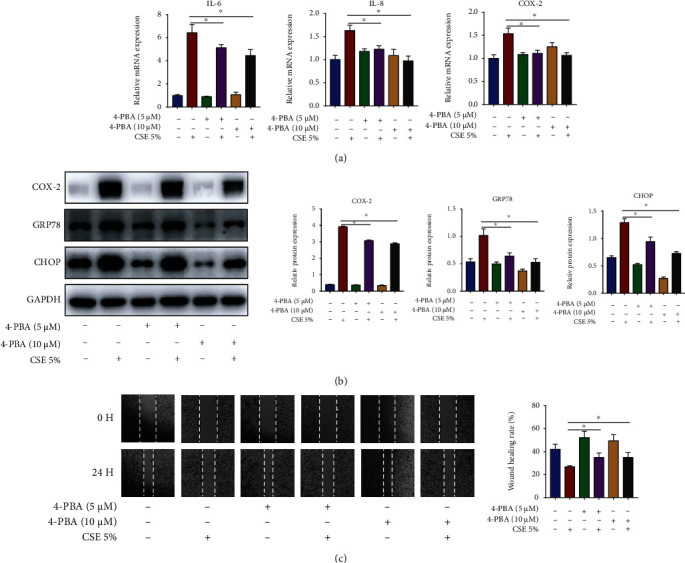
ER stress regulates inflammation and cell migration in CSE-stimulated HBECs. HBECs were pretreated with 4-PBA (5 *μ*M or 10 *μ*M) for 1 h and then exposed to 5% CSE for 24 h. (a) RT-qPCR assessment of IL-6 and IL-8 along with COX-2 mRNA contents in HBECs. (b) Western blot images and quantification of COX-2, GRP78, and CHOP protein contents in HBECs. (c) Image analysis of cell migration. ^∗^*P* < 0.05; *n* = 3.

To study the relationship among inflammation, ER stress, and cell migration, we used ER stress inhibitor 4-PBA to treat HBECs prior to 5% CSE stimulation. It was observed that, in CSE-stimulated HBECs, 4-PBA decreased the mRNA contents of IL-6 and IL-8 along with COX-2 (Figure 4(a)), reduced the protein contents of COX-2, GRP78, and CHOP (Figure 4(b)), and promoted cell migration (Figure 4(c)). The above results illustrated that ER stress played a pivotal upstream role in CSE-induced airway epithelial injury by promoting inflammation and inhibiting cell migration.

### 3.5. IRE1*α* Is the Target Gene of miR-150-5p

**Figure 5:**
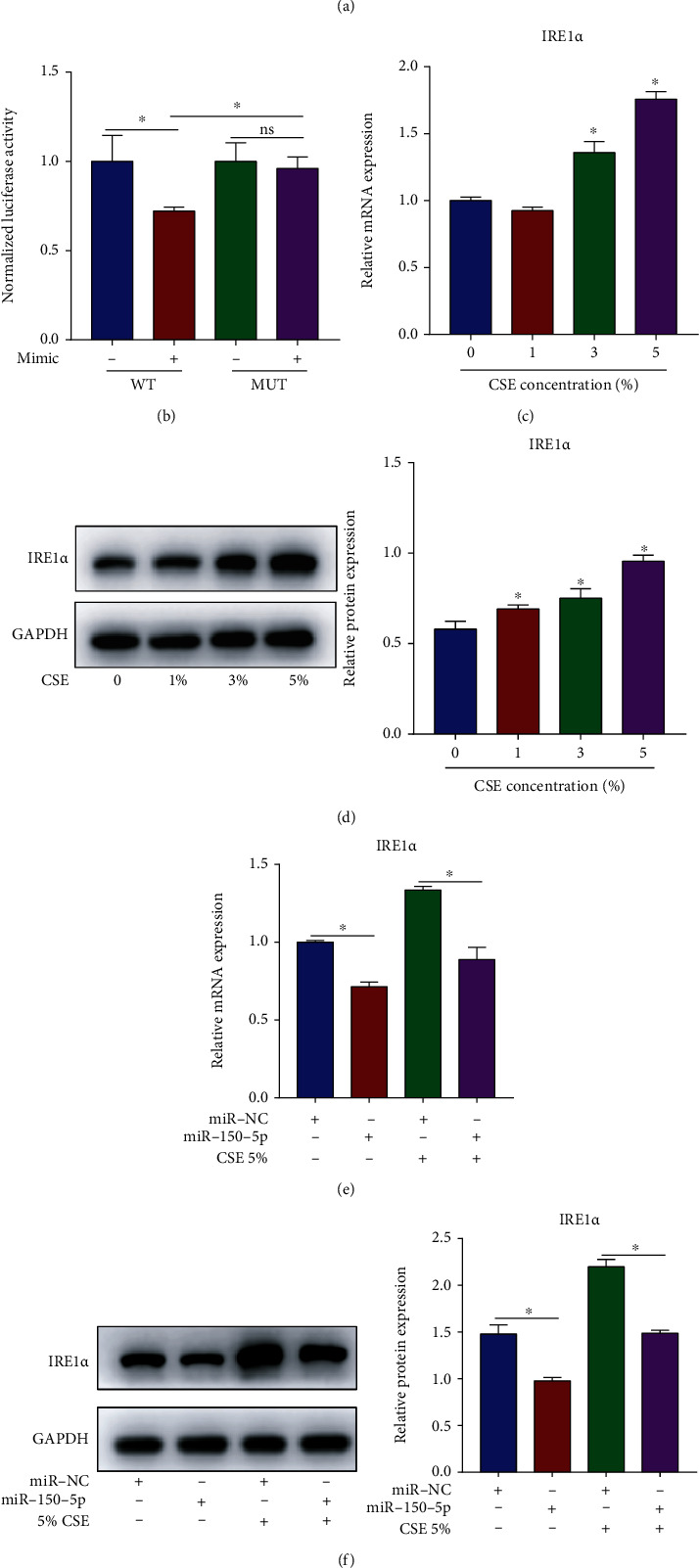
IRE1*α* is the target gene of miR-150-5p. (a) The putative miR-150-5p binding sequence in the 3′-UTR of IRE1*α* was predicted by the RNA22 database. (b) HBECs were transfected with IRE1*α* WT or MUT vector together with miR-150-5p mimic or NC mimic prior to 5% CSE stimulation, and luciferase activity was measured. HBECs were exposed to diverse levels of CSE (1, 3, and 5%) for 24 h. (c) IRE1*α* mRNA content assessment via RT-qPCR and (d) protein content assessment via western blot. HBECs were transfected with miR-150-5p mimic or NC mimic prior to 5% CSE stimulation. (e) IRE1*α* mRNA content assessment via RT-qPCR and (f) protein content assessment via western blot. ^∗^*P* < 0.05; *n* = 3.

In accordance with the RNA22 database, IRE1*α* was selected as a possible miR-150-5p target gene (Figure 5(a)). Luciferase reporter assay was adopted to verify the miR-150-5p-IRE1*α* target relationship. In contrast with NC mimic, miR-150-5p mimic remarkably suppressed relative luciferase activity in the IRE1*α* WT group but did not affect that in the IRE1*α* MUT group (Figure 5(b)). In addition, we detected IRE1*α* expression in CSE-stimulated HBECs. The contents of IRE1*α* mRNA and protein were increased concentration-dependently (Figures 5(c) and 5(d)) but were remarkably declined after miR-150-5p mimic transfection (Figures 5(e) and 5(f)). The above findings revealed that miR-150-5p targeted IRE1*α* in CSE-stimulated HBECs.

### 3.6. IRE1*α* Aggravates Inflammation and ER Stress and Inhibits Cell Migration in CSE-Stimulated HBECs

**Figure 6:**
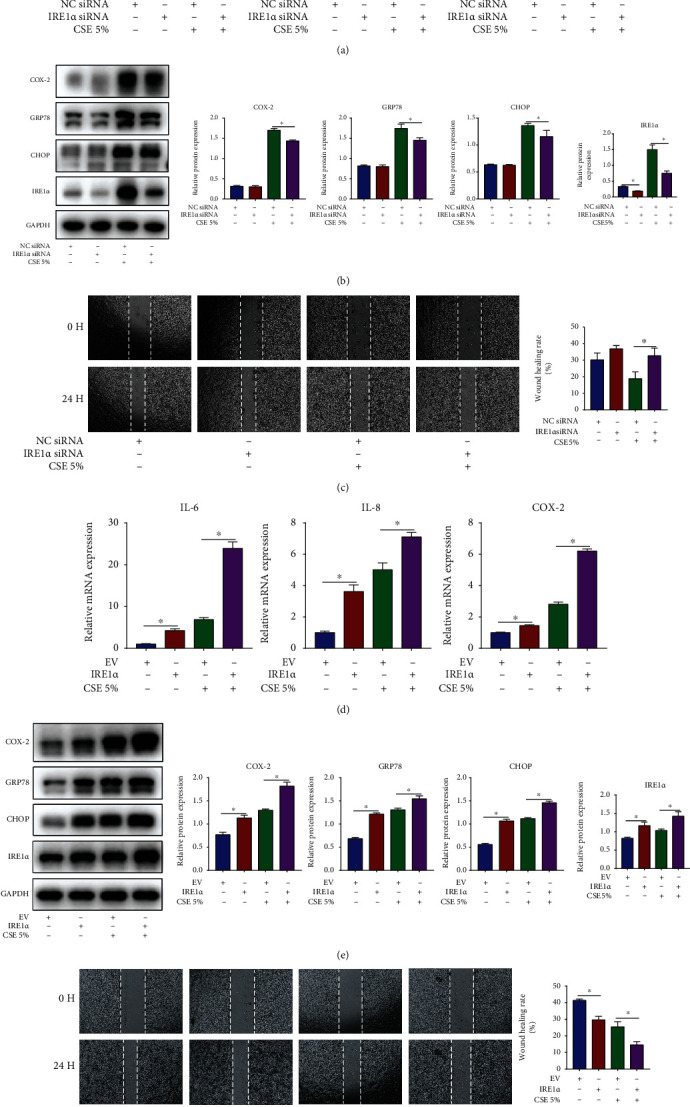
IRE1*α* aggravates inflammation and ER stress and inhibits cell migration in CSE-stimulated HBECs. HBECs were transfected with IRE1*α* siRNA or NC siRNA and then exposed to 5% CSE for 24 h. (a) RT-qPCR assessment of IL-6 and IL-8 along with COX-2 mRNA contents in HBECs. (b) Western blot images and quantification of COX-2, GRP78, CHOP, and IRE1*α* protein contents in HBECs. (c) Image analysis of cell migration. HBECs were transfected with IRE1*α* overexpression vector or empty vector (EV) and then stimulated with or without 5% CSE for 24 h. (d) RT-qPCR assessment of IL-6 and IL-8 along with COX-2 mRNA contents in HBECs. (e) Western blot images and quantification of COX-2, GRP78, CHOP, and IRE1*α* protein contents in HBECs. (f) Image analysis of cell migration. ^∗^*P* < 0.05; *n* = 3.

To determine whether IRE1*α* participates in CSE-induced airway epithelial injury, IRE1*α* siRNA or overexpression vector was used to downregulate or upregulate IRE1*α* expression in HBECs prior to 5% CSE stimulation, respectively. In CSE-stimulated HBECs, IRE1*α* siRNA remarkably decreased the mRNA contents of IL-6 and IL-8 along with COX-2 (Figure 6(a)), reduced the protein contents of COX-2, GRP78, and CHOP (Figure 6(b)), and promoted cell migration (Figure 6(c)). Conversely, overexpression of IRE1*α* elevated the mRNA contents of IL-6 and IL-8 along with COX-2 (Figure 6(d)), increased the protein contents of COX-2, GRP78, and CHOP (Figure 6(e)), and suppressed cell migration (Figure 6(f)). It is important to note that, overexpression of IRE1*α* alone, without CSE stimulation, could boost the contents of IL-6, IL-8 along with COX-2, GRP78, and CHOP and suppress cell migration. The above results indicated that the upregulation of IRE1*α* contributed to CSE-induced airway epithelial injury.

### 3.7. miR-150-5p Regulates CSE-Induced Inflammation, ER Stress, and Cell Migration in HBECs by Targeting IRE1*α*

**Figure 7:**
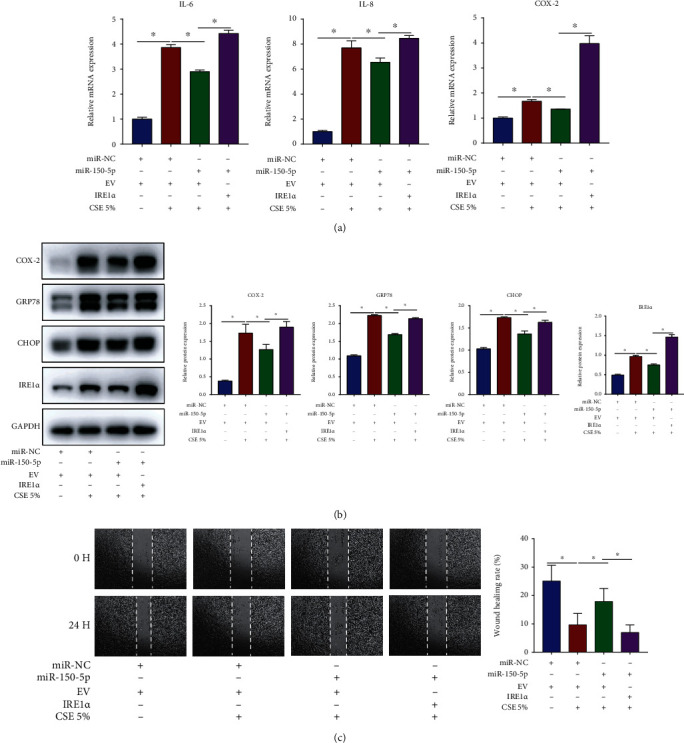
miR-150-5p regulates CSE-induced inflammation, ER stress, and cell migration in HBECs by targeting IRE1*α*. HBECs were transfected with miR-150-5p mimic or NC mimic together with IRE1*α* overexpression vector or empty vector (EV) prior to 5% CSE stimulation. (a) RT-qPCR assessment of IL-6 and IL-8 along with COX-2 mRNA contents in HBECs. (b) Western blot images and quantification of COX-2, GRP78, CHOP, and IRE1*α* protein contents in HBECs. (c) Image analysis of cell migration. ^∗^*P* < 0.05; *n* = 3.

Further determining whether miR-150-5p regulated CSE-induced airway epithelial injury via targeting IRE1*α*, we treated HBECs with miR-150-5p mimic or NC mimic together with IRE1*α* overexpression vector or empty vector (EV) before 5% CSE stimulation. As shown in Figures 7(a)–7(c), in CSE-stimulated HBECs, miR-150-5p mimic significantly decreased the mRNA contents of IL-6 and IL-8 along with COX-2, reduced the protein contents of COX-2, GRP78, and CHOP, and promoted cell migration. The aforesaid effects of miR-150-5p were, however, reversed by IRE1*α* overexpression. Altogether, these results illustrated that miR-150-5p modulated inflammation, ER stress, and cell migration by targeting IRE1*α* expression in CSE-stimulated HBECs.

### 3.8. ROS Regulates CSE-Induced miR-150-5p Expression in HBECs

**Figure 8:**
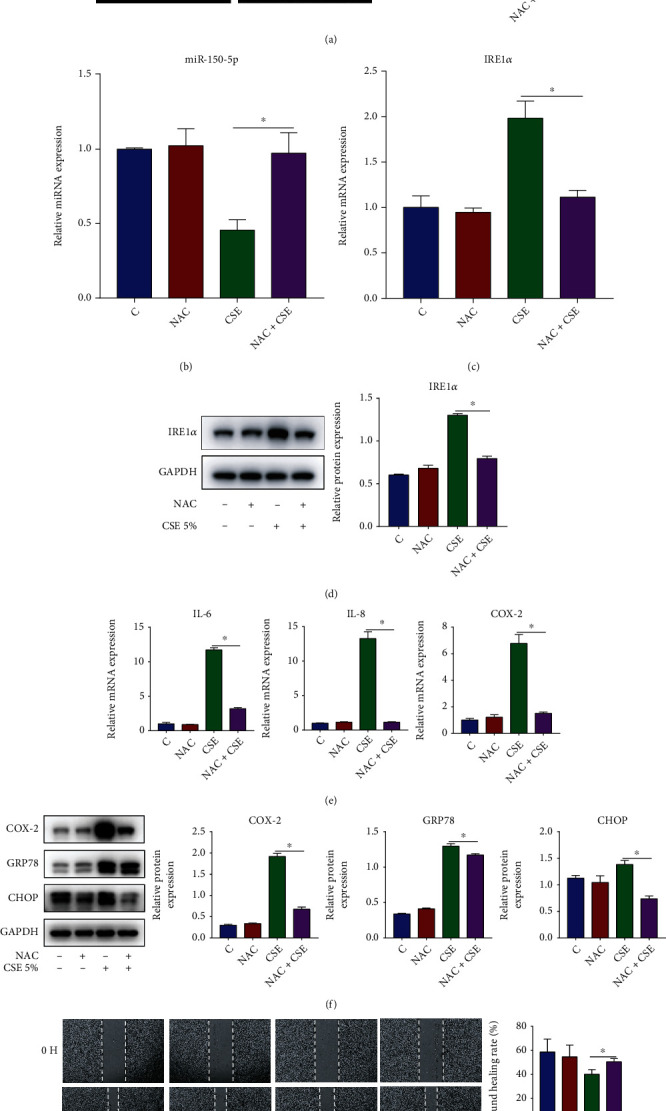
NAC prevents CSE-induced miR-150-5p downregulation and IRE1*α* upregulation. HBECs were pretreated with 5 mM NAC for 1 h and then exposed to 5% CSE for 24 h. (a) DCFH-DA analysis of intracellular ROS production. (b) RT-qPCR assessment of miR-150-5p expression in HBECs. (c) RT-qPCR assessment of IRE1*α* mRNA contents in HBECs. (d) Western blot images and quantification of IRE1*α* protein contents in HBECs. (e) RT-qPCR assessment of IL-6 and IL-8 along with COX-2 mRNA contents in HBECs. (f) Western blot images and quantification of COX-2, GRP78, and CHOP protein contents in HBECs. (g) Image analysis of cell migration. ^∗^*P* < 0.05; *n* = 3.

To clarify whether ROS regulated CSE-induced miR-150-5p downregulation, we treated HBECs with NAC, a ROS scavenger, prior to 5% CSE stimulation. It was observed that NAC treatment inhibited the production of intracellular ROS (Figure 8(a)) and reversed CSE-induced miR-150-5p downregulation (Figure 8(b)) and IRE1*α* upregulation (Figures 8(c) and 8(d)). Additionally, NAC treatment also decreased the mRNA contents of IL-6 and IL-8 along with COX-2 (Figure 8(e)), reduced the protein contents of COX-2, GRP78, and CHOP (Figure 8(f)), and promoted cell migration in CSE-stimulated HBECs (Figure 8(g)).

### 3.9. miR-150-5p Inhibits IRE1*α* Expression and Ameliorates CS-Induced Inflammation and ER Stress In Vivo

**Figure 9:**
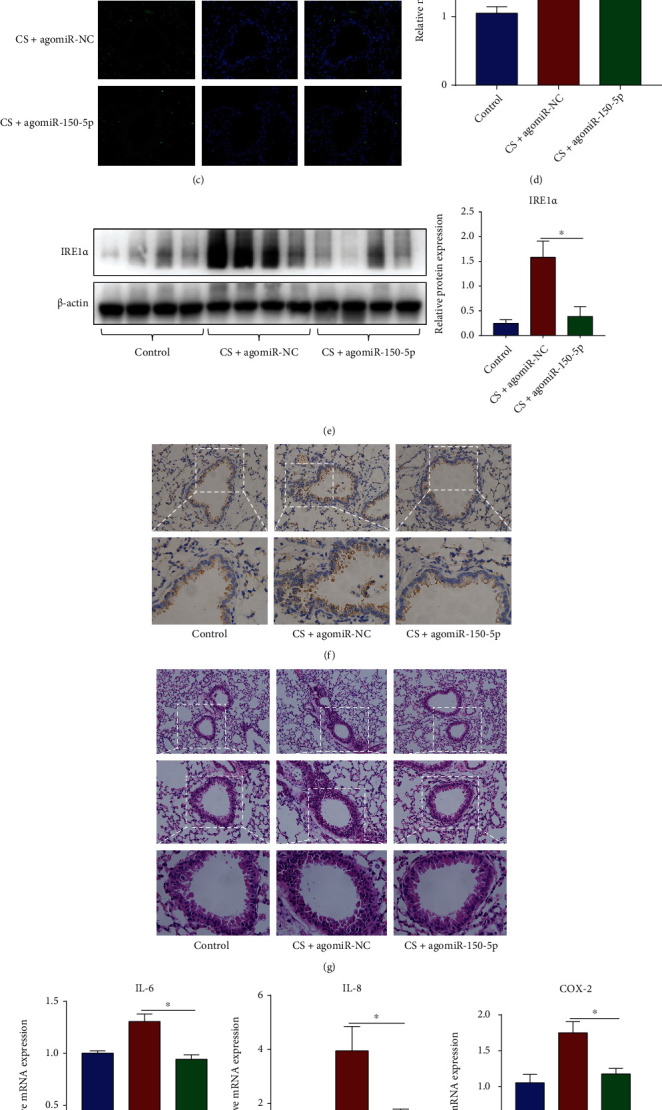
miR-150-5p inhibits IRE1*α* expression and ameliorates CS-triggered inflammation and ER stress in vivo. The 3-day CS-exposed mouse model was constructed, and agomiR-150-5p, as well as agomiR-NC, was administered nasally into mice before CS exposure. (a) Diagram of the experimental workflow. (b) RT-qPCR assessment of miR-150-5p expression in lung tissues from different groups. (c) FISH images of miR-150-5p staining in lung tissue sections (original magnification, ×400). (d) RT-qPCR assessment of IRE1*α* mRNA contents in lung tissues from different groups. (e) Western blot images and quantification of IRE1*α* protein contents in lung tissues from different groups. (f) IHC images of IRE1*α* staining in lung tissue sections (original magnification, ×200 (upper) and ×400 (down)). (g) H&E images of pathological changes in lung tissue sections (original magnification, ×100 (upper), ×200 (middle), and ×400 (down)). (h) RT-qPCR assessment of IL-6 and IL-8 along with COX-2 mRNA contents in lung tissues from different groups. (i) Western blot images and quantification of COX-2, GRP78, and CHOP protein contents in lung tissues from different groups. ^∗^*P* < 0.05; *n* = 4‐6.

For further evaluating miR-150-5p's effects on CS-induced COPD, we chose the 3-day CS-exposed mouse model for study. miR-150-5p agomiR was administered nasally to mice to upregulate miR-150-5p before exposure to CS (Figure 9(a)). In contrast with the controls, CS exposure reduced miR-150-5p expression, but miR-150-5p agomiR reversed the downregulation (Figure 9(b)). Consistent with the above differences, FISH analysis also identified miR-150-5p was predominantly localized to airway epithelium (Figure 9(c)). RT-qPCR, western blot, and IHC analysis exhibited that CS exposure increased IRE1*α* expression, while agomiR-150-5p reversed the upregulation of IRE1*α* induced by CS exposure (Figures 9(d)–9(f)). As illustrated in Figures 9(g)–9(i), miR-150-5p agomiR attenuated CS-induced lung inflammatory cell infiltration, reduced the contents of IL-6, IL-8 along with COX-2, GRP78, and CHOP in the lung tissues. The above results further confirmed that miR-150-5p is protective to CS-induced COPD via inhibition of IRE1*α* expression.

## 4. Discussion

Herein, we sought to assess the role along with the potential mechanisms of miR-150-5p in CS-induced COPD. The results illustrated that miR-150-5p was decreased in the plasma of COPD and AECOPD patients and related to disease diagnosis, disease severity, and lung function. In CSE-stimulated HBECs and CS-exposed mice, miR-150-5p attenuated inflammation and ER stress and promoted cell migration, which was achieved by inhibiting IRE1*α* expression. Additionally, ROS regulated CSE-induced miR-150-5p downregulation and its downstream effects. Our results highlighted plasma miR-150-5p as a protective biomarker and the ROS/miR-150-5p/IRE1*α* axis as a therapeutic target in CS-induced COPD.

Increasing studies have shown that monitoring of circulating miRNA expression is of great significance in the early diagnosis and prognostic evaluation of diseases. In our study, we first searched for miR-150-5p expression in different samples and found that miR-150-5p was highly abundant in the blood, illustrating its possible as a circulating biomarker. Further analysis of the miRNA dataset GSE31568 identified that blood miR-150-5p was downregulated in COPD patients. A number of diseases have been linked to circulating miR-150-5p downregulation. For example, plasma miR-150-5p was reduced in acute myeloblastic leukemia and was associated with the diagnosis of this disease [10]. Another study identified that low plasma miR-150-5p expression could distinguish patient groups with different types of colon diseases, as well as differentiate patients with advanced cancer from those with benign diseases [11]. Besides, in colorectal cancer, serum exosomal miR-150-5p was found to be an independent prognostic indicator [30]. In a previous investigation, low blood miR-150-5p expression was linked to poor survival in COPD patients [15]. Herein, we also illustrated that miR-150-5p was reduced in the plasma of COPD and AECOPD patients and was related to disease diagnosis, disease severity, and lung function. This is consistent with the findings of Pottelberge et al. [31], who found that genes associated with lung function impairment were significantly enriched in miR-150-5p binding sites in a genome-wide association study, suggesting an essential role for miR-150-5p in the initial link of smoking-induced lung function decline. Furthermore, in mice exposed to acute and chronic CS, miR-150-5p downregulation in the plasma was positively linked with that in the lung, providing support for miR-150-5p as an early and specific biomarker for COPD.

Airway epithelial injury is central to COPD pathogenesis [32, 33], and thus, we used HBECs with CSE stimulation as an in vitro model. Consistent with the data derived from COPD patients and CS-exposed mice, CSE reduced miR-150-5p expression in HBECs in a concentration along with time-dependent approach. Inflammation leads to airway epithelial injury and is essential for COPD development. Moreover, ER stress has also been shown to participate in CS-induced COPD. As reported, CS exposure was associated with early induction of ER stress [34]. Inhibiting ER stress could alleviate CS-induced airway inflammation and emphysema [35]. In addition, cell migration is a critical process for the recovery of intact epithelium following airway epithelial injury [36]. Our study exhibited that miR-150-5p prevented CSE-induced airway epithelial injury by inhibiting inflammation and ER stress and promoting cell migration, which was further confirmed in the 3-day CS-exposed mouse model. A previous study reported that mesenchymal cell-derived miRNA-150-5p exosome had therapeutic potential in rheumatoid arthritis [37]. Recently, adipose-derived mesenchymal stem cell (ADMSC-) originated miR-150-5p extracellular vesicles were identified to attenuate hepatic fibrosis [38]. Hopefully, miRNA-150-5p may be clinically applied in the future.

According to recent studies investigating the relationship among inflammation and ER stress along with cell migration, ER stress has the ability to directly initiate inflammatory pathways, such as NF-*κ*B signaling [39], JNK signaling [40], and inflammasome activation [41]. However, the effects of ER stress on cell migration vary with the tissue and/or microenvironment [42, 43]. Generally, ER stress contributes to cell migration in a tumoral context but impairs cell migration in a nontumoral context. To date, whether ER stress regulates CSE-induced inflammation and cell migration in HBECs remains unidentified. Our results showed that 4-PBA pretreatment reduced CSE-induced inflammation and promoted cell migration, suggesting a critical upstream role for ER stress in CSE-induced airway epithelial injury.

Mechanistically, IRE1*α* was established as the target gene of miR-150-5p. IRE1*α* is one of the sensor proteins in ER stress signaling and determines the state and fate of cell [44]. When ER stress occurs, it triggers the adaptive UPR transcription program, and in severe cases, it initiates processes such as inflammation and apoptosis. A recent study discovered that IRE1*α* affected cell migration by directly interacting with filamin A, which was independent of its canonical role as an ER stress transducer [45]. As of yet, not much knowledge is to investigate IRE1*α* effects on COPD pathogenesis. In our research premise, we demonstrated that the upregulation of IRE1*α* promoted inflammation and ER stress and inhibited cell migration in CSE-induced HBECs. Notably, a previous study reported that IRE1*α* could degrade miR-150-5p in the development of fibrotic diseases [46]. This is consistent with our results that 4*μ*8C, a specific inhibitor of IRE1*α*, could reverse CSE-induced miR-150-5p downregulation. Notably, 4*μ*8C alone could upregulate miR-150-5p expression (seen in Supplementary Figure 2). Thus, it may be reasonable to speculate that in COPD, low miR-150-5p expression resulted in IRE1*α* upregulation, which may in turn further reduce miR-150-5p expression, thus forming a vicious circle. Targeting the miR-150-5p/IRE1*α* cascade may therefore be a promising therapeutic strategy for COPD.

In addition, we explored the regulatory mechanisms behind miR-150-5p downregulation in CSE-stimulated HBECs. Oxidative stress is an important mechanism underlying CS-induced impairment of airway epithelial cells and is also an important pathological feature of COPD [47, 48]. Activating the antioxidant system or increasing endogenous antioxidants levels has been evaluated as potential treatments for COPD [49, 50]. As reported, there exists a complex crosstalk between ROS and miRNAs [51]. Some miRNAs are expressed in response to ROS, which may in turn be targeted by miRNAs. Oxidized miR-184 by ROS enabled it to misrecognize of Bcl-xL along with Bcl-w as its targets and thus promoted apoptosis [52]. The differential expression of miR-1 mediated by ROS contributed to distinct pathologic heart conditions [53]. Notably, recent research found that hyperoxia decreased miR-150-5p expression through ROS in lung epithelial cells [54]. In our study, ROS scavenger NAC treatment prevented CSE-induced miR-150-5p downregulation and upregulation of its downstream target gene IRE1*α*. Moreover, NAC treatment also inhibited CSE-induced HBECs inflammation and ER stress and promoted cell migration. Our results thus confirmed that ROS mediated CSE-induced miR-150-5p downregulation and its downstream effects. Put another way, our research further confirmed that inhibiting oxidative stress could protect against COPD, and this effect may be achieved partly by increasing expression of protective miRNAs such as miR-150-5p.

## 5. Conclusion

In summary, plasma miR-150-5p was a protective biomarker in CS-induced COPD. Mechanistically, ROS mediated CSE-induced miR-150-5p downregulation, and miR-150-5p reduced CSE or CS-induced inflammation and ER stress and promoted cell migration by targeting IRE1*α* in vitro along with in vivo. These findings may provide a new perspective for diagnosing and treating COPD.

## Data Availability

The datasets generated during and/or analyzed during the current study are available from the corresponding author on reasonable request.

## Supplementary Materials

Supplementary Materials Supplementary Figure 1: miR-150-5p has high abundance expression and was decreased in COPD blood. Supplementary Figure 2: IRE1*α* degrades the expression of miR-150-5p. Supplementary Table 1: characteristics of the study population. Supplementary Table 2: management strategies of AECOPD patients in hospital. Supplementary Table 3: primers sequences used for RT-qPCR.
