# Supplementary material for: ROS-Responsive miR-150-5p Downregulation Contributes to Cigarette Smoke-Induced COPD via Targeting IRE1α
Source: Oxid Med Cell Longev. 2022 May 5;2022:5695005. doi: 10.1155/2022/5695005 (PMC9098354; doi:10.1155/2022/5695005)
Supplement: Supplementary Materials — Supplementary Figure 1: miR-150-5p has high abundance expression and was decreased in COPD blood. Supplementary Figure 2: IRE1α degrades the expression of miR-150-5p. Supplementary Table 1: characteristics of the study population. Supplementary Table 2: management strategies of AECOPD patients in hospital. Supplementary Table 3: primers sequences used for RT-qPCR. [file 5695005.f1.docx]

**Supplementary Figure 1 miR-150-5p has high abundance expression and was decreased in COPD blood.** (a) The expression of miR-150-5p in different samples was searched through the YM500v2 database. (b) The expression of miR-150-5p in the blood of COPD patients (n=70) compared to normal controls (n=24) was analyzed from GSE31568 dataset. Results are shown as mean ± SEM. *, *P* < 0.05.


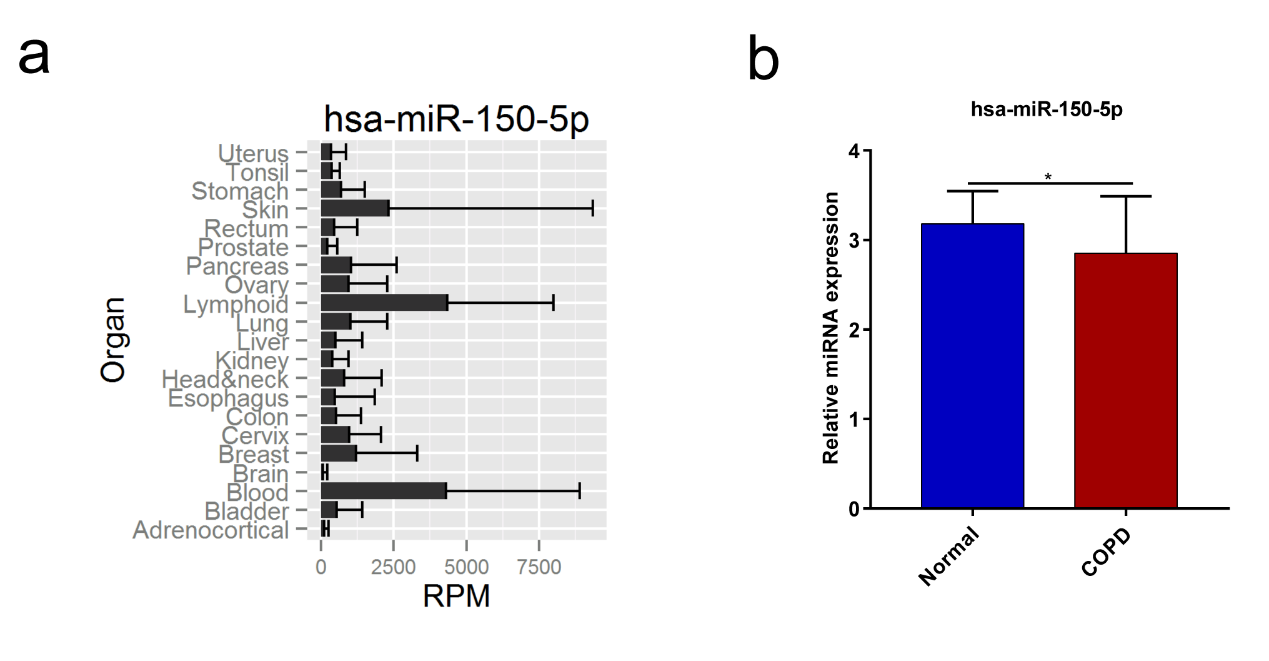


**Supplementary Figure 2 IRE1α degrades the expression of miR-150-5p.** HBECs were pretreated with 4μ8C (10 μM, 50 μM and 100 μM) for 1 h prior to 5% CSE stimulation, and the miR-150-5p expression was detected by RT-qPCR. Results are shown as mean ± SEM. *, *P* < 0.05; n = 3.


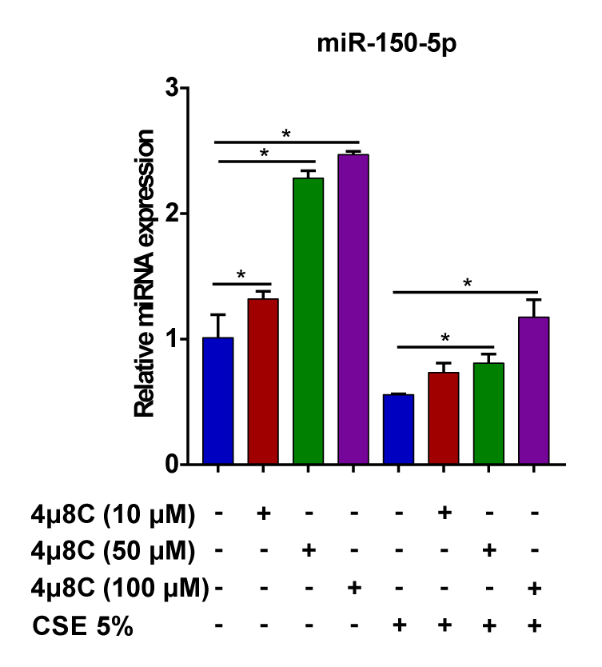


**Supplementary Table 1:** Characteristics of the study population.

| **Parameters** | **Non-smoker** | **Smoker** | **COPD** | **AECOPD** |
| --- | --- | --- | --- | --- |
| Male (number) | 7 | 10 | 20 | 13 |
| Age (years) | 61.83 ± 3.35 | 62.9 ± 1.79 | 66.74 ± 1.83 | 70.54 ± 2.56 |
| BMI (kg/m^2^) | 24.33 ± 0.89 | 25.02 ± 0.94 | 22.76 ± 0.50 | 22.8 ± 1.10 |
| FEV_1_ | 2.89 ± 0.30 | 2.72 ± 0.08 | 1.37 ± 0.13 | 1.09 ± 0.18 |
| FEV_1_%pred | 105 ± 7.73 | 90.93 ± 3.57 | 46.11 ± 3.57 | 42.07 ± 5.84 |
| FEV_1_/FVC | 82.42 ± 2.35 | 76.7 ± 1.65 | 50.89 ± 2.63 | 45.23 ± 2.94 |
| Smoking index (pack-years) | NA | 27.27 ± 3.37 | 53.29 ± 4.76 | 46.43 ± 5.64 |

**Supplementary Table 2:** Management strategies of AECOPD patients in hospital.

| **Variable** |  | **Number (%)** |
| --- | --- | --- |
| Respiratory support | Oxygen therapy | 9 (69.23) |
|  | Ventilator support | 4 (30.77) |
| Pharmacologic treatment | Inhaled anticholinergics | 8 (61.54) |
|  | Inhaled corticosteroid (ICS) | 8 (61.54) |
|  | Oral corticosteroid | 3 (23.08) |
|  | Intravenous corticosteroid | 8 (61.54) |
|  | Intravenous doxofylline | 8 (61.54) |
|  | Antibiotics | 13 (100) |
|  | N-Acetylcysteine (NAC) | 5 (38.46) |
|  | Expectorants | 12 (92.31) |
| Hospital stay (days) |  | 11.42 ± 1.21 |

**Supplementary Table 3.** Primers sequences used for RT-qPCR.

| **Genes** | **Forward primer** | **Reverse primer** |
| --- | --- | --- |
| **(human)** |  |  |
| miR-150-5p | TCTCCCAACCCTTGTACCAGTG |  |
| IL-6 | ACATCCTCGACGGCATCTCA | CCAGTGCCTCTTTGCTGCTT |
| IL-8 | TCTCTTGGCAGCCTTCCTGA | TTTCTGTGTTGGCGCAGTGT |
| COX-2 | AATCTGGCTGCGGGAACACAAC | TGTCTGGAACAACTGCTCATCACC |
| GRP78 | CCGAGGAGGAGGACAAGAAGGAG | ACACGCCGACGCAGGAGTAG |
| CHOP | CTGCTTCTCTGGCTTGGCTGAC | CTCCCTTGGTCTTCCTCCTCTTCC |
| IRE1α | CCAGCACCAGCAGTTCCAGAAG | CCGTCCTGAGCCGTGTCTCC |
| **(mouse)** |  |  |
| miR-150-5p | TCTCCCAACCCTTGTACCAGTG |  |
| IL-6 | TGGGACTGATGCTGGTGACA | ACAGGTCTGTTGGGAGTGGT |
| IL-8 | GATGCTCCATGGGTGAAGGC | AAGCTTCATTGCCGGTGGAA |
| COX-2 | GGTGCCTGGTCTGATGATGTATGC | CCTATGAGTATGAGTCTGCTGGTTTGG |
| IRE1α | TTCCACCAGCCTCTATGCCTCTC | ATCACACACTCTCCTTTGTCTCCAATG |
